# Enzyme inactivation induced by thermal stabilization in highland barley and impact on lipid oxidation and aroma profiles

**DOI:** 10.3389/fnut.2023.1097775

**Published:** 2023-03-02

**Authors:** Qianna Zheng, Zheng Wang, Feiyang Xiong, Guoquan Zhang

**Affiliations:** College of Food Science and Engineering, Northwest A&F University, Xianyang, Shaanxi, China

**Keywords:** highland barley, thermal stabilization, enzymatic activities, volatile compounds, PLS-DA

## Abstract

Thermal stabilization is efficient for slowing lipid degradation and prolonging the shelf life of highland barley, but the impacts of different thermal stabilized treatments on highland barley and possible chemical reactions remain unclear. The effects of thermal stabilization treatments (bake, far-infrared, fry, microwave and steam) on the enzymes, lipids and aroma profiles of highland barley flour (HBF) were investigated in this study. Thermal stabilization significantly decreased the contents of ash and GABA. Baked HBF exhibited the lowest fatty acid value and peroxide value. Untreated HBF had higher lipase and lipoxygenase activities and fried mostly inactivated these enzymes. All thermal stabilization treatments increased the catalase activities and fried showed the higher level. Thus, fried might be an effective method to stabilize the HBF. The high temperatures during stabilizing triggered the complex reactions, leading to the loss of some volatile compounds, and in the meantime the formation of others such as furans and aldehydes. These productions contributed to the unique aroma profiles of different HBFs. Furthermore, a chemometric approach was used to analyze the changes of thermal stabilized treated HBFs and to identity six key volatile compounds, which provided important knowledge on possible chemical reactions caused by thermal stabilization. Overall, these results provide the theoretical basis for the wider application of thermal stabilization technologies in highland barley processing.

## 1. Introduction

Highland barley, the most distinctive crop produced in Qinghai Tibet Plateau, which has gained much attention in recent years due to its unique nutritional values (1). Highland barley is abundant in β-glucan, arabinoxylan, dietary fiber, vitamins, polyphenols, and GABA, which endow highland barley with the functions of preventing cancer and controlling cholesterol and blood glucose levels (2).

Highland barley has a thick and organizational outer layer structure, including seed coat, pericarp and aleurone layer. The main bioactive components in highland barley, such as *β*-glucan and phenolic compounds, are mainly distributed in the outer layer structure (3). Due to the short of advanced milling technology and the demand on nutrition, most of the aleurone layer and germ are retained in the highland barley flour. The lipase existing in aleurone layer and germ may induce depredate of lipids through enzymatic oxidation and hydrolysis, resulting in the deterioration of the flour and the short shelf life (4). The oxidative rancidity of lipids causes the deterioration of product sensory quality and the loss of nutritional properties (5). Therefore, inactivation of enzyme activities may be an appropriate strategy for preventing or slowing lipid degradation and prolonging the shelf life.

Many researchers have utilized high temperature to destroy the structure of enzyme, resulting in the inactivation of enzyme activity, the good storage stability and better flavor. In general, the thermal stabilization technologies mainly include bake, far-infrared, microwave, fry and steam and other technologies such as superheated steam and ultrasound. Baking, frying and steaming are traditional methods of cereal processing, and they have the advantages of simple operation and low equipment cost (6). Zhao et al. (7) found that roasting treatment of highland barley decreased the lipase activity by 81.1% with the moisture content 20%. Microwave and infrared heating are commonly recognized as hopeful alternative methods to traditional methods because of the short time taken and an efficient energy provided (8). Rose et al. (9) determined the effectiveness of steam and microwave treatments of stabilization in whole wheat flour, the lipase activity decreased by 93 and 96% of microwave and steam treatments only for 1 min. Dang et al. (10) found that the moisture content and lipase activity of the whole grain highland barley flour after thermal treatment remained at a low level, and the fatty acid value, peroxide value, and malondialdehyde value increased. It has been reported that superheated steam processing improved storage properties of highland barley by improving the stability of lipid (11). Li et al. (12) examined the enzymolysis kinetics of rice protein pretreated with ultrasound. They found that the ultrasound assisted alkali caused the less active enzyme for lower enzyme loading, resulted in the initial rate declination of enzyme catalytic reaction kinetics. The contribution of ultrasound effects to improve the enzymolysis reaction rate constant was more significant at lower temperatures than at higher temperatures (13).

The volatile compounds of highland barley are complex and diverse, which has an important impact on the sensory quality of highland barley products and directly determines the acceptance of consumers (14). Thermal stabilization has a certain impact on highland barley flavor due to the enzymatic oxidation, maillard reaction and thermally induced lipid oxidation will change the flavor. In other words, aldehydes and ketones produced in the lipid degradation will interact with amino acids or reducing sugars which produced in maillard reaction (15). At present, the research on volatile compounds changes during processing is mostly concentrated on other cereals such as wheat, rice and oat, while little in highland barley. Lampi et al. (16) studied the stability of heat-treated oat grains, and extensive lipid oxidation occurred once the temperature reached 130°C. Wei et al. (17) investigated the aroma deteriorations of rice bran by infrared radiation heating. They found that the contents of esters, aldehydes, and phenols in the infrared radiation-treated group were significantly higher than those in the control group.

In summary of the literatures, the majority of existing literatures on the properties of highland barley by thermal stabilization have been performed with a single processing technology, whereas there are few with respect to the comparison of bake, far-infrared, microwave, fry and steam. The effects of thermal treatments on the structure and physicochemical characteristics of HBF are currently being investigated from the standpoint of the enzyme-destroying effect, and all these studies have concentrated on the effect of thermal stabilization on the structure of starch and protein. Nonetheless, no study has further contrastive analysis of the lipids and flavor properties of HBF after these thermal stabilization technologies inactivate enzymes. Only by clarifying these properties and possible chemical reaction can the appropriate treatment method be selected in accordance with the demands of various products for raw materials.

Overall, highland barley thermal stabilization provokes the occurrence of complex biochemical reactions, leading to the changes of nutrients, enzymes activities and volatile flavor compounds. At present, the researches on the thermal stabilization of highland barley flour mainly focus on the structural properties, and few studies have clarified and compared the effects of different thermal stabilization techniques on enzyme activity and storage stability. At the meantime, this paper preliminarily speculated the possible chemical reactions in the thermal stabilization technology and marked the key compounds, which is of great significance to further study and better apply the thermal stabilization technology to highland barley products for improving storage stability and reducing the undesirable odor.

## 2. Materials and methods

### 2.1. Materials

Highland barley grains were provided by Tibet Academy of Agriculture and Animal Husbandry Sciences. The moisture content of highland barley was adjusted to 5% after sieving and removing impurities. After different thermal stabilization, five kinds of highland barley flour (HBF) with 4% pearling were obtained using a cyclone mill (FW-400 AD, XINBODE, China).

### 2.2. Thermal stabilization processing

About 1 kg of HBF was prepared for experiment, of which 200 g was used for the control group and each experiment group, respectively. The HBF was steamed, baked, fried, microwaved and infrared as follows.

#### 2.2.1. Steam processing

Distilled water was put into a household electric cooker (MZ-ZG28W4–001, Media, China) and the water was heated. When the water was boiled and the hot steam was filled in the cooker, and the HBF was put on the steam grid. The HBF was steamed for 30 min at 900 w and then taken out.

#### 2.2.2. Baked processing

The HBF was treated in hot air oven (101-1AB, Taisite, Tianjin, China) for 30 min at 155°C.

#### 2.2.3. Fried processing

A household electric oven (C22-WT2202, Media, Guangdong Province, China) was used and the oven was adjusted to 100°C at 900 W. After 30 min, the HBF was taken out.

#### 2.2.4. Microwaved processing

The HBF was put near the center position in microwave oven (X3-233A, Media, Guangdong Province, China) for 3 min at 560 W.

#### 2.2.5. Infrared processing

A far-infrared electric oven (KWS1530X-H7R, Galanz, Guangdong Province, China) was used and the HBF was treated for 3 min at 900 w, 210°C. The non-treated HBF was used as control.

### 2.3. Proximate composition

The HBF samples were estimated for their moisture (44–01), crude protein (N × 6.25) (46-11A), crude fat (30–10), ash (08–01), and mineral elements (40–70) were analyzed according to AACC methods. Dietary fiber, total starch, amylose content and β-glucan contents were determined using the megazyme mixed-linkage assay kit (Megazyme International Ltd., Ireland). *γ*-aminobutyric acid (GABA) was measured as described by Kitaoka (18).

### 2.4. Analysis of fatty acid value

Firstly, the HBF (10 g) was mixed with 50 ml petroleum ether, shook for 10 min, and filtered and collected the filtrate. The filtrate (25 ml) was mixed with 75 ml 50% ethanol solution and titrated with 0.01 M KOH. Added five drops of phenolphthalein, until a pink color appeared and persisted for 30 s. The sample was replaced with petroleum ether for a blank test. The phenolphthalein (1%, w/v) was used as an indicator and the fatty acid value (mg KOH/100 g) was calculated according to the Eq (1).


$$\text{Fatty acid value}=\left(V_{1}-V_{2}\right)\times c\times 56.1\times \frac{50}{25}\times \frac{100}{m\times \left(100-w\right)}\;\left(1\right)$$

where V_1_ = volume of sample titration (mL), V_2_ = volume of blank titration (mL), c = normality of titrant (mol/L), m = weight of sample (g), and w = moisture of sample (%).

### 2.5. Analysis of peroxide value and catalase activity

Peroxide value (PV) was determined according to AOCS method (19). The catalase (CAT) activity was determined according to GB/T 5522–2008 (20).

### 2.6. Determination of lipase activity

Lipase (LIP) activity of HBF was measured as follows: about 2 g HBF was mixed with 1 ml pure oil and phosphate buffer solution (5 ml, pH 7.4, 0.1 M) and the mixture was allowed to react at 30°C for 24 h. After that, 50 ml of a mixture of ethanol and ether was added, shook well and stood for 1–2 min. This was followed by filtration and pipetting 25.0 ml of filtrate. Phenolphthalein indicator was added and titrated with 0.05 M potassium hydroxide solution until it was reddish and colorless for 30 s.

### 2.7. Determination of lipoxygenase activity

Lipoxygenase (LOX) activity was determined according to method described by Cato (21) with slight modification.

To prepare the linoleic acid substrate: 0.5 ml Tween-20 was dissolved in borate buffer (10 ml, pH 9.0, 0.05 M) and mixed well. 0.5 ml linoleic acid was added dropwise to emulsion, and then NaOH (1.3 ml, l M) solution was added until the solution was clarified. Finally, 90 ml of the borate buffer was added, and the volume was fixed to 200 ml with distilled water, and 1 M HCl was used to adjust the pH to 7.0.

To extract the enzyme, 0.5 g HBF was added with 2.5 ml phosphate buffer (0.05 M, pH 7.5) and incubated at 4°C for 30 min. After that, the mixture was centrifuged with a speed of 8,000 r/min at 4°C for 10 min.

Reaction system: Sodium acetate buffer (9.5 ml, 0.05 M, pH 5.6) and 0.3 ml linoleic acid substrate were mixed with 60 μl enzyme extract, and then the absorbance was measured at 234 nm using a spectrophotometer (TU-1810, Puxi General Instrument Co., Ltd., Beijing, China). The enzyme extract in the reaction system was replaced by the inactivated enzyme extract as the substrate control. One LOX activity unit was defined as an increase in absorbance of 0.01 at 234 nm within 1 min.

### 2.8. Determination of volatile compounds

Volatile compounds were extracted with a headspace solid phase micro-extraction (HS-SPME) coated with a divinylbenzene/carboxen/polydimethylsiloxane (DVB/CAR/PDMS, 50/30 μm) film and analyzed on a gas-chromatography/mass spectrometry (GC/MS) (QP2010 Ultra, Shimadzu, Japan). Two grams of HBF were weighed in a 20 ml headspace vial. The SPME sampling was performed by inserting the SPME fiber into the headspace with heating (45°C) and continuous stirring for 50 min. After extraction, it was injected into the GC–MS and desorbed the volatile compound at 250°C for 5 min. The volatile compounds were separated on a Restek fused silica capillary column (30 m × 0.25 mm × 0.25 μm) and helium was used as the carrier gas with a constant flow rate of 1 ml/min in a splitless injection mode. The temperature program was set as follows: The oven temperature was held at 40°C for 3 min, then increased to 120°C at a rate of 4°C min and finally increased to 240°C at a rate of 6°C/min. The MS detector was conducted by electron impact (EI) source at the following conditions: electron energy 70 eV; ionization temperature, 230°C; the transfer line temperature was 220°C; scan range 35–500 m/z. The reference mass spectra of National Institute of Standards and Technology (NIST) was used for the identification of the volatiles by comparing their programmed temperature retention indices (RI) in two different phases of gas chromatograph column, molecular weights, and mass fragmentation patterns. The relative contents of each volatile compound were quantified by peak area normalization. The analysis was carried out in triplicate.

### 2.9. Data processing and multivariate analyses

Statistical analyses were performed using Minitab 8.0. The mean values were compared *via* one-way and Tukey’s *post hoc* test was used to detect significant differences of different stabilized HBF samples (*p* ≤ 0.05). Results were presented as the means ± standard deviations of triplicate samples. The figures were plotted using Origin 8.0.

To investigate the effect of thermal stabilized treatments on the integrated data of volatiles compounds (VOC), a regression-based supervised classification technique, which called partial least squares discriminant analysis (PLS-DA) was applied. Score-plots were generated using the PLS-DA model to visualize the contributions and changes of VOCs. Variable importance in projection (VIP) scores were calculated to estimate the importance of each variable in the PLS projection. Variables with VIP >1 were the most relevant variable for explaining *Y*-variables (SIMCA 14.1 software).

## 3. Results and discussion

### 3.1. Effect of thermal stabilization on proximate composition

There were great diversities in proximate composition (moisture, ash, amylose, GABA and mineral elements) of HBF affected by thermal stabilization (Table 1). When steamed, the moisture content of HBF was higher than that of the control group, while other treatment groups were lower than that. In all thermal stabilized treated samples, a decreasing tendency of the ash contents was observed, thereby improving the color and smoothness of HBFs. Table 1 showed that fat, protein, total dietary fiber and β-glucan contents of thermal stabilized treated groups did not show any significant variation. Microwave treatment significantly decreased the total starch content whereas increased the amylose content. The *γ*-aminobutyric acid content was reduced significantly after microwave and infrared treatment. Different stabilizing treatments had significant effects on calcium content of HBF, which may be due to the oxidation reaction of calcium. The statistical analysis showed significant increase in zinc content of microwaving, frying and baking, whereas it is not significant for infrared and steaming treatments, which may be related to the state of binding and solubility of minerals; (22). Iron content was significantly decreased in steaming treatment group, while the other treatments significantly increased the iron content. Copper content of steamed HBF was significantly lower than the control group but there was no difference after other treatments, which may be attributed to the oxidation reaction occurred in humid air. Baking treatment significantly reduced phosphorus content of HBF, while steamed, microwaved and infrared technologies significantly increased phosphorus content. Selenium content of fried HBF was significantly higher than the raw HBF and other treatments had no significant effects.

**Table 1:** Proximate composition of the highland barley flour.

Sample	Moisture (%)	Ash (%)	Fat (%)	TS (%)	Protein (%)	Amylose content (%)	TDF (%)	*β*-glucan (%)	GABA (%)	Calcium (mg/kg)	Zinc (mg/kg)	Iron (mg/kg)	Copper (mg/kg)	Phosphorus (mg/100 g)	Selenium (mg/kg)	Total mineral (mg/kg)
Raw	9.06 ± 0.04b	2.34 ± 0.35a	1.92 ± 0.32a	69.09 ± 0.45a	10.74 ± 0.09a	18.17 ± 1.08bc	19.72 ± 1.20a	4.03 ± 1.24a	1.98 ± 0.10a	439 ± 1d	16.00 ± 0.14c	39.65 ± 0.35c	4.72 ± 0.04a	347 ± 3c	0.0056 ± 0.0004b	533.58 ± 1.53d
Infrared	8.31 ± 0.97b	0.66 ± 0.11b	2.26 ± 0.24a	70.73 ± 6.07a	10.35 ± 0.00a	23.01 ± 2.11b	20.35 ± 1.02a	5.67 ± 0.77a	1.22 ± 0.16c	419 ± 1e	16.30 ± 0.14c	41.90 ± 0.42b	4.74 ± 0.08a	370 ± 1b	0.0057 ± 0.0001b	518.89 ± 1.55e
Baked	5.49 ± 0.31c	0.72 ± 0.23b	2.25 ± 0.27a	63.06 ± 1.07ab	10.92 ± 0.27a	20.02 ± 2.11bc	20.39 ± 0.15a	4.94 ± 0.95a	1.77 ± 0.17ab	468 ± 1b	19.40 ± 0.28a	43.70 ± 0.14a	4.92 ± 0.06a	78.65 ± 1d	0.0064 ± 0.0001ab	543.391 ± 0.276c
Microwaved	5.60 ± 0.73c	0.80 ± 0.11b	2.20 ± 0.16a	53.68 ± 1.02b	10.59 ± 0.33a	35.72 ± 1.06a	18.89 ± 0.33a	4.83 ± 0.39a	1.48 ± 0.04bc	457 ± 0c	17.45 ± 0.21b	43.60 ± 0.57a	4.92 ± 0.01a	389 ± 2a	0.0065 ± 0.0002ab	561.826 ± 0.552b
Fried	3.61 ± 0.32c	0.30 + 0.06b	2.21 ± 0.13a	64.09 ± 0.46ab	11.04 ± 0.80a	22.21 ± 1.05bc	20.09 ± 0.16a	4.02 ± 1.11a	1.98 ± 0.10a	543 ± 0a	17.90 ± 0.14b	44.40 ± 0.28a	4.85 ± 0.08a	347 ± 4c	0.0076 ± 0.0006a	644.853 ± 0.771a
Steamed	13.11 ± 0.02a	0.50 ± 0.00b	2.58 ± 0.06a	65.63 ± 1.91a	9.85 ± 0.016a	16.65 ± 1.08c	20.81 ± 0.35a	3.85 ± 0.14a	1.72 ± 0.05ab	376 ± 2f	15.85 ± 0.35c	36.15 ± 0.07d	4.17 ± 0.04b	373 ± 1b	0.0053 ± 0.0003b	468.93 ± 1.81f

TS, total starch; TDF, total dietary fiber; GABA, *γ*-aminobutyric acid. Data are the mean ± standard deviation of triplicates. Different lowercase letters within a column indicate statistical differences (*p* ≤ 0.05).

### 3.2. Effect of thermal stabilization on fatty acid value and peroxide value

**Figure 1:**
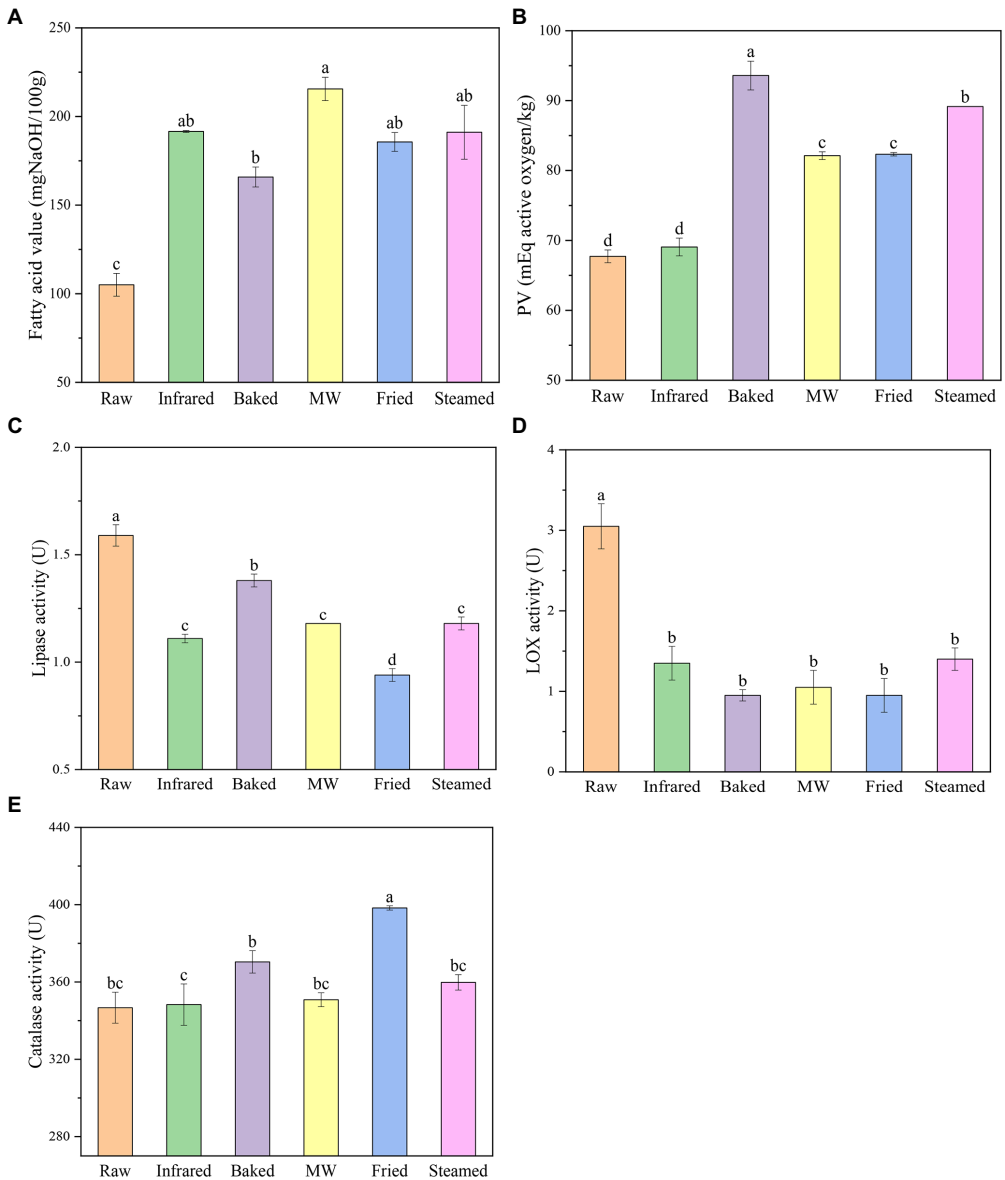
Fatty acid value **(A)**, peroxide value **(B)**, lipase **(C)**, lipoxygenase **(D)**, and catalase **(E)** activities of highland barley flour. Different lowercase letters indicate significantly different at level *p* ≤ 0.05.

Changes of lipid properties were usually used for monitoring the quality deterioration of flour because lipid was more prone to degradation rapidly than that of protein and starch (23). Fatty acids value was used as an indicator of the degree of lipid hydrolysis (24). Figure 1A portrayed the free fatty acid content changes of HBF with respect to different treatments. The fatty acid value of raw HBF was 23.11 mg/100 g and the HBFs after infrared, baked, microwaved, fried, and steamed were 23.73, 20.67, 27.00, 23.21, and 23.79 mg/100 g, respectively. This result indicated that hydrolytic rancidity of HBF not obviously occurred during thermal stabilization. Rose et al. (9) also reported that microwave treatment or steam treatment had no obvious effect on the fatty acid value of wheat flour.

The peroxide value (PV) provides a measure of the initial oxidative rancidity of lipid during storage (25). Peroxide values of infrared and baked HBF had no obvious difference with the raw HBF (Figure 1B). The peroxide value of raw HBF was 0.34 meq/kg. After microwaved, fried and steamed, the PV of HBF were 1.77, 1.81, and 0.94 meq/kg, respectively, indicating that lipid oxidation (both enzymatic and non-enzymatic) occurred during stabilization treatments of HBF. The reason might be microwaved, fried and steamed treatments induced deformation of outer layers structure and disruption of endosperm cell walls, and thus increased the fat-soluble components (1).

### 3.3. Inactivation of lipase and lipoxygenase

Lipases hydrolyzed the ester bonds of triacylglycerols, contributing to the aging flavor, so it was important to inactivate the lipase for longer storage. All thermal stabilized treatments effectively reduced the LIP activity (Figure 1C). The raw HBF had the highest LIP activity (1.59 mg/g). The infrared, baked, microwaved, fried, steamed HBF lipase activities were significantly reduced by 30.2, 13.2, 25.8, 40.9, and 25.8%, respectively. The different reduction percentage of LIP activity employed thermal stabilization was expected, since previous report have indicated that microwave, steam, infrared, and other thermal stabilization exert a good effect on lipase inactivation (6). Our results suggested that the lipase activity was more stable to bake treatment than the other treatments. Meanwhile, fried HBF was more efficient at decreasing lipase activity than others, which meant fried treatment mostly enhanced the HBF storage stability by reducing enzymatic oxidation reactions. Whereas LIP could not be completely inactivated which might be explained by the fact that LIP was relatively stable compared with other enzymes and the activity was too low to be reduced. This result was kept in with the reports of Li et al. (26), who found that the inactivation rates of lipase in microwaved or roasted HB grain and flour were 50 or 60%.

The LOX activities declined sharply for all the treatments, and the decrease rates were more apparent compared with lipase (Figure 1D). This result was consistent with Xu et al. (27), who found that lipase activity was more heat-stable than the lipoxygenase. There were no significant difference among different thermal stabilized treatments (31–46%). Baked and fried were found to be most effective in inactivating lipoxygenase (*p* > 0.5). These results are in accord with recent studies indicating that the LOX decreased rate was 60.2% when the flours were subjected to treatments of thermal infrared rays (28). Moreover, the result clearly outlined again that fried followed by other thermal stabilization reduced the occurrence of enzymatic oxidation reactions.

### 3.4. Effect of thermal stabilization on catalase activity

Hydrogen peroxide in living organisms was catalyzed by catalase to form oxygen and water, which protected organisms from damage caused by reactive oxygen species (29). Study has shown that the catalase activity was closely related to the vigor of seeds and could be used as a judge index of grain freshness (30). The changes in catalase activity of HBF were shown in Figure 1E. Catalase activities were found to be the highest in fried HBF and the increase rate was 14.8%. The other treatments increased the catalase activity but the results were not significant. The possible reason might be that the differences in moisture and temperature in different treatments. Gili et al. (31) reported that water activity improved the sensitivity of enzymes to heat, and water could be used as a good transfer medium of heat.

### 3.5. Determination of volatile compounds

Using the SPME-GC–MS method, there were 36 volatile compounds detected in HBFs (Table 2). In terms of categories, VOCs were divided into eight classes including alcohols, aldehydes, alkanes, esters, hydrocarbon, ketones, furans, and others. These compounds are generally considered to be derived from the photosynthesis and metabolism of proteins, free amino acids, carbohydrates, triglycerides or their derivatives, as well as vitamins and minerals (32).

**Table 2:** Comparisons of the detected VOCs in highland barley flour by SPME-GC-MS.

Compounds	Formula	CAS	Molecular weight	Retention index	Highland barley flour relative content (%)					
					Raw	Infrared	Baked	Microwaved	Fried	Steamed
Aldehydes										
Acetaldehyde	C2H4O	75-07-0	44	408	2.183 ± 0.661c	13.747 ± 1.592a	7.840 ± 0.820b	ND	ND	ND
2-Methylpropanal	C4H8O	78-84-2	72	543	0.5350 ± 0.4010bc	0.2548 ± 0.0534c	0.8262 ± 0.0292b	0.3988 ± 0.0279bc	0.6359 ± 0.1374bc	1.7607 ± 0.1702a
Butanal	C4H8O	123-72-8	72	607	ND	0.2518 ± 0.0581a	ND	ND	ND	ND
3-Methylbutanal	C5H10O	590-86-3	86	643	0.3820 ± 0.2130b	ND	ND	ND	0.7611 ± 0.1165b	4.370 ± 0.491a
2-Methylbutanal	C5H10O	96-17-3	86	643	0.2065 ± 0.1542c	0.4235 ± 0.0849bc	0.6127 ± 0.0414b	0.5126 ± 0.0327b	0.5158 ± 0.0166b	2.695 ± 0.145a
Pentanal	C5H10O	110-62-3	86	707	ND	ND	ND	ND	ND	1.8204 ± 0.09a
Hexanal	C6H12O	66-25-1	100	806	20.27 ± 0.99bc	26.40 ± 1.35b	ND	16.59 ± 1.70c	18.37 ± 6.93bc	36.55 ± 1.10a
Nonanal	C9H18O	124-19-6	142	1,104	2.811 ± 0.469a	ND	ND	ND	ND	ND
Alcohols										
2,5-Cyclooctadien-1-ol	C8H12O	10,054-74-7	124	1,112	0.7354 ± 0.0702a	ND	ND	ND	ND	ND
Ethanol	C2H6O	64-17-5	46	463	5.965 ± 0.778d	18.574 ± 0.995a	15.277 ± 0.797b	13.678 ± 0.196b	8.164 ± 0.625c	13.421 ± 0.388b
1-Penten-3-ol	C5H10O	616-25-1	86	671	0.6386 ± 0.0877a	ND	ND	ND	ND	ND
1-Pentanol	C5H12O	71-41-0	88	761	2.729 ± 0.280b	ND	ND	ND	ND	4.337 ± 0.445a
1-Heptacosanol	C27H56O	2004-39-9	396	2,948	0.4895 ± 0.1353a	ND	ND	ND	ND	ND
P-Mentha-1,8-Dien-7-ol	C10H16O	536-59-4	152	1,261	2.771 ± 0.719a	ND	ND	ND	1.504 ± 0.130b	ND
1-Hexanol	C6H14O	111-27-3	102	860	3.067 ± 0.733a	ND	ND	ND	1.7914 ± 0.1478b	ND
Hydrocarbon										
Dimethyl-Diazene	C2H6N2	503-28-6			ND	ND	ND	14.951 ± 0.639a	ND	ND
Benzene	C6H6	71-43-2	78	680	3.284 ± 0.196b	2.854 ± 0.170bc	2.567 ± 0.100c	1.484 ± 0.382d	2.989 ± 0.185bc	6.507 ± 0.200a
Toluene	C7H8	108-88-3	92	794	1.797 ± 0.041d	2.705 ± 0.131bc	3.142 ± 0.304b	1.735 ± 0.052d	2.454 ± 0.129c	5.819 ± 0.238a
(*E*)-2-Octen-1-ol	C8H16O	18,409-17-1	128	1,067	ND	ND	ND	ND	ND	0.7242 ± 0.1125a
d-Limonene	C10H16	5,989-27-5	136	1,018	ND	1.563 ± 0.067b	1.651 ± 0.054b	1.316 ± 0.377b	ND	3.772 ± 1.222a
Others										
Dimethyl peroxide	C2H6O2	690-02-8	62	372	ND	2.197 ± 0.222a	ND	ND	ND	ND
Dimethyl sulfide	C2H6S	75-18-3	62	471	0.4154 ± 0.1339a	ND	ND	ND	ND	ND
Ammonium acetate	C2H7NO2	631-61-8	77	630	ND	6.269 ± 0.709a	ND	ND	ND	ND
Acetic acid	C2H4O2	64-19-7	60	576	7.222 ± 0.769a	ND	ND	ND	ND	ND
2,4-Di-tert-butylphenol	C14H22O	96-76-4	206	1,555	10.29 ± 0.01a	ND	ND	ND	ND	ND
Alkanes										
2,2,4,6,6-Pentamethyl heptane	C12H26	13,475-82-6	170	981	17.18 ± 1.58 cd	7.696 ± 0.104e	30.18 ± 0.20a	19.92 ± 0.68c	23.76 ± 2.59b	13.70 ± 1.03d
2,2,4,4-Tetramethyloctane	C12H26	62,183-79-3	170	1,045	1.475 ± 0.0829b	ND	2.125 ± 0.386a	0.7854 ± 0.0572c	ND	ND
Dodecane	C12H26	112-40-3	170	1,214	0.6004 ± 0.065b	ND	ND	ND	0.4958 ± 0.025b	1.413 ± 0.265a
Tetradecane	C14H30	629-59-4	198	1,413	ND	ND	ND	ND	ND	0.3832 ± 0.0323a
Esters										
Ethyl acetate	C4H8O2	141-78-6	88	586	16.83 ± 1.70e	21.28 ± 0.92d	36.902 ± 0.93b	32.69 ± 1.338c	42.8 ± 1.75a	ND
2-Methyl ethoxyacetate	C5H10O3	0-00-0	118	761	ND	ND	ND	ND	ND	3.683 ± 1.143a
Formic acid hexyl ester	C7H14O2	629-33-4	130	981	ND	1.703 ± 0.351b	ND	0.6552 ± 0.0312c	ND	3.697 ± 0.488a
Furans										
Tetrahydrofuran	C4H8O	109-99-9	72	589	ND	ND	ND	ND	ND	0.5381 ± 0.0786a
2-Pentylfuran	C9H14O	3,777-69-3	138	1,040	0.7486 ± 0.0675a	ND	ND	ND	ND	ND
Ketones										
2-Butanone	C4H8O	78-93-3	72	555	0.6314 ± 0.0266a	ND	ND	0.2521 ± 0.0316b	0.3146 ± 0.0723b	ND
Acetoin	C4H8O2	513-86-0	88	717	ND	ND	ND	ND	ND	1.047 ± 0.300a

ND, not detected or value below the detection threshold. Different lowercase letters in the same row are significantly different (*P* ≤ 0.05).

**Figure 2:**
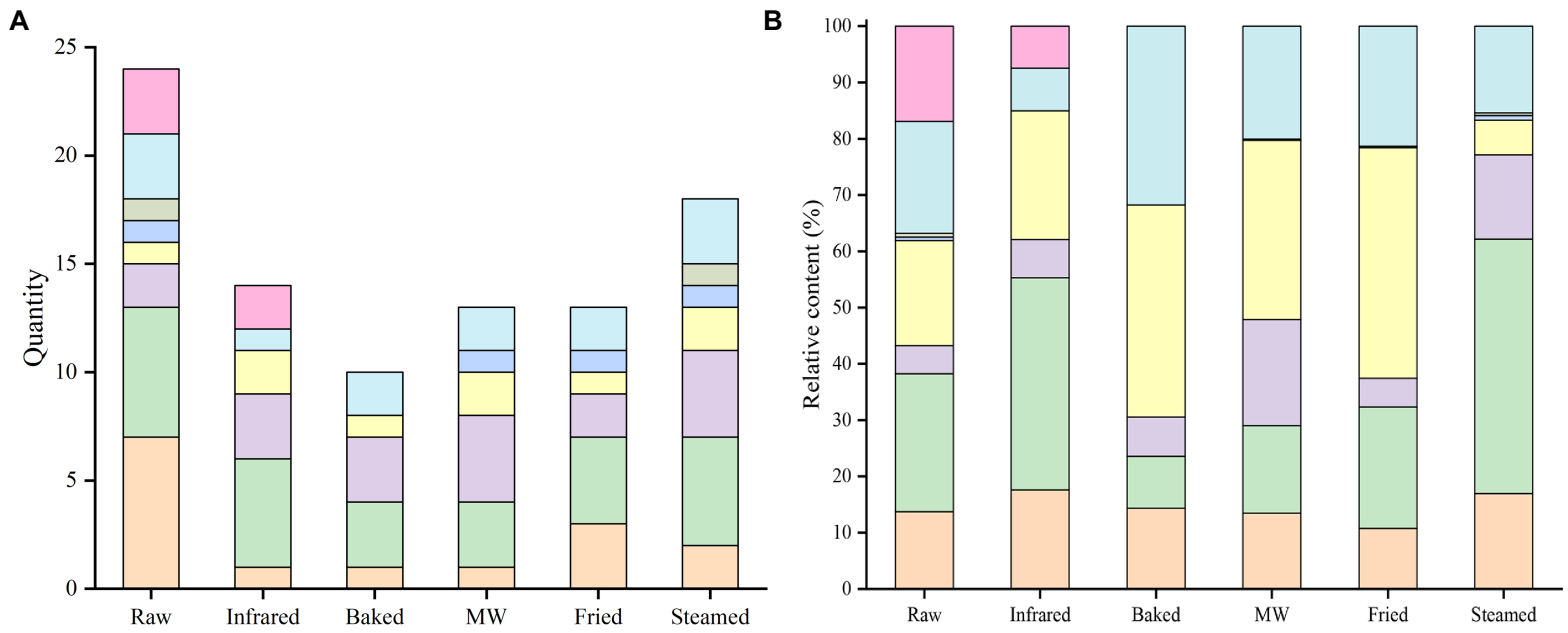
Quantity **(A)** and relative content **(B)** of volatile compounds in highland barley flour. 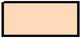 Alcohols, 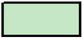 Aldehydes, 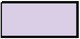 Hydrocarbon, 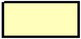 Esters, 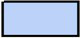 Ketones, 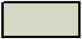 Furans, 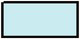 Alkanes, and 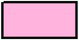 Others.

The numbers of VOCs in raw and stabilized processed samples were different (Figure 2A), including 24 in raw HBF (seven alcohols, six aldehydes, three alkanes, two hydrocarbon, and six other compounds), 14 in infrared HBF (five aldehydes, three hydrocarbons, two esters, and four other compounds), 10 in baked HBF (three aldehydes, three hydrocarbons, two alkanes, and two other compounds), 13 in microwaved HBF (four hydrocarbons, three aldehydes, two alkanes, two esters, and two other compounds), 13 in fried HBF (four aldehydes, three alcohols, two hydrocarbons, two alkanes, and two other compounds), and 18 in steamed HBF (five aldehydes, four hydrocarbons, three alkanes, two alcohols, two esters, and two other compounds).

Aldehydes were the dominant compounds of raw (Figure 2B), infrared and steamed HBF which may be due to the low sensory threshold (33). The straight-chain aldehydes such as hexanal were derived from the linoleic acid oxidation, which are considered safe and could be used as flavor substances in food (34). It was worth mentioning that steamed treatment remarkable increase the strecker aldehydes such as 3-Methyl butanal and 2-Methyl butanal. Strecker degradation is one of side reactions of the Maillard reaction. The first step of Maillard reaction was a condensation involving an amino acid and a reducing sugar with a carbonyl group. The result of this condensation was a not thermostable Amadori compound, which could be demolished and produced the rearranged sugars due to the prolonged storage periods (35). The sugars then could be split into dicarbonyl and degraded the amino acids, leading to the deamination and decarboxylation, which were called Strecker degradation. The Strecker aldehydes were closely related to flavor formation (36). The formation of these compounds was triggered during steaming and the process seemed to have a combined effect of Maillard reaction and non-enzymatic oxidation. These aldehydes gave the unique aroma of fat, grass, caramel of HBF. The highest contents in baked, microwaved and fried HBF were esters (Figure 2B), which might come from the raw material or the esterification reaction of alcohols and acids and contribute to the sweet and fruity aroma of the HBF samples, which indicated that thermal stabilization changed the mainly aromatic substances. As occurs in aldehydes, steamed HBF presented the highest contents than others. The volatile compound present at the highest level in all treatments was hexanal followed by acetaldehyde. This finding was consistent with some studies that reported the hexanal was the major compound in cereal flour (37). As occurs in esters, fried showed the highest value, and ethyl acetate was the dominant VOC.

### 3.6. Partial least squares-discriminant analysis

**Figure 3:**
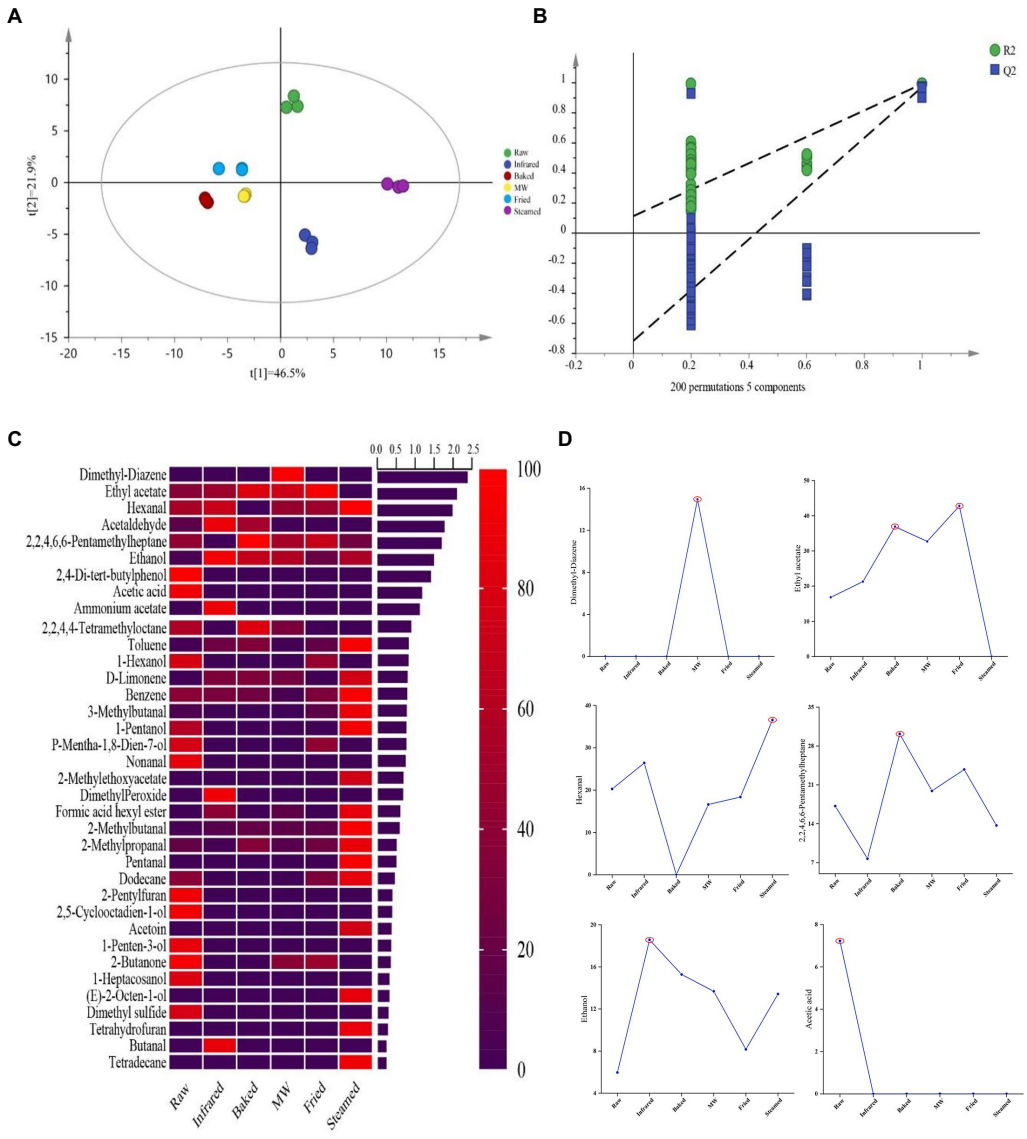
Partial least squares discriminant analysis (PLS-DA) score plots **(A)**, permutations plots **(B)**, heatmaps **(C)**, and discriminant markers **(D)** analysis of the volatile aroma compounds identified in highland barley flour. The treatment that the compound was selected as a discriminant marker was marked with red color circle.

To deeply highlight the differences of aroma compounds in raw and stabilized HBF, the PLS-DA was applied to investigate the contributions and changes of aroma compounds of stabilized treatment. The differences among six HBF samples had been distinctly identified in the PLS-DA score plots as shown in Figure 3A, which exhibited a total variance of 68.4% (PLS1, 46.5%; PLS2, 21.9%). The permutations plot helped to assess the risk that the current PLS-DA model was spurious (Figure 3B). The idea of this validation was to compare the goodness of fit (R2 and Q2) of the original model with the goodness of fit of several models based on data where the order of the Y-observations has been randomly permuted, while the X-matrix has been kept intact. Result demonstrated that the PLS-DA model was valid and not overfitted, therefore validated the VIP values obtained from this model (Figure 3B).

A heatmap was generated (Figure 3C) based on the contents of VOCs. Taking the estimated concentrations of the identified 36 VOCs as variables, each variable was normalized by sum. The row comparison showed the distribution frequency of each substance in the six samples. Color coding was graded on the basis of the scale with the relative intensity increasing from low (purple) to high (red) for one volatile compound. Comparing the different treatments, raw HBF displayed higher content of 2, 5-cyclooctadien-1-ol, dimethyl sulfide, 2-butanone, acetic acid, 1-penten-3-ol, 1-hexanol, p-mentha-1, 8-dien-7-ol, nonanal, 1-heptacosanol and 2, 4-di-tert-butylphenol. The profile of VOCs was changed after treatments. For example, dimethyl peroxide, butanal and ammonium acetate were only detected in infrared group and dimethyl-diazene only in microwaved HBF. Most VOCs were produced after steaming such as 2-methylethoxy acetate, tetrahydrofuran, pentanal, (E)-2-octen-1-ol, tetradecane, et al.

Variable importance in projection scores were calculated to select compounds driving the classification and that were significantly affected by the treatments. All the variables presenting a VIP > 1 were considered relevant for the discrimination of raw samples HBF from thermal stabilized processed HBFs. According to the literature reported and the VIP values, 6 common VOCs were selected as key VOCs for further analysis (Figure 3C). The treatment that the compound was selected as a discriminant marker was marked with red color circle (Figure 3D). Among these VOCs, there were two hydrocarbons (Dimethyl-diazene and 2, 2, 4, 6, 6-Pentamethyl heptane), an esters (ethyl acetate), an aldehydes (hexanal), an alcohols (ethanol) and an acids (acetic acid). The hydrocarbons could be generated by decarboxylation and cleavage of carbon chains of fatty acids, thus they might be produced by the oxidation and decomposition of HBF during high temperature treatment (38). Dimethyl-diazene showed the highest content in microwaved and baked group. These VOCs were considered could provide fatty, fruity and green aroma characters depending on their concentration. Hexanal was the basic product of linoleic acid oxidation. The oxidation of linoleic acid produced the 13-hydroperoxide, which could cleaved to hexanal and gave HBF the aroma of fatty, grass, and caramel (39). Acetic acid was a volatile component found in almost all crops, and its content decreased significantly after all thermal stabilization. Alcohols had a higher threshold, which contributed to the aroma of light, sweet, fruity, floral, and other mellow notes and infrared significantly increased the ethanol content. Compared with other HBFs, fried and baked HBFs showed the higher contents of ethyl acetate, which gave them the characteristic flavor of sweet and fruity.

## 4. Conclusion

This study gave a comprehensive insight into the effect of thermal stabilization technologies on the enzymes, lipids and volatile compounds of highland barley. The results demonstrated that thermal stabilization had no obvious effects on the contents of fat, protein, total dietary fiber and *β*-glucan. The activities of lipase and lipoxygenase in HBF were significantly decreased by the thermal stabilization and frying seemed to be the most effective method to inactivate the enzyme activities. While the catalase activity was improved in all groups which might be caused by the sensitivity differences of enzymes. Baking significantly inhibited the increase of fatty acid value in HBF. Five types of thermal stabilized technologies had obviously different effects on the aroma profiles. Aldehydes acted as the dominant compounds of raw, infrared and steamed HBF while the highest relative contents in baked, microwaved and fried HBF were esters. Non-enzymatic oxidation could be promoted and played a key role when the thermal stabilized treatments were applied. After thermal stabilizing, six volatile compounds (Dimethyl-diazene, 2, 2, 4, 6, 6-pentamethyl heptane, ethyl acetate, hexanal, ethanol, and acetic acid) were identified through PLS-DA. These results of this study could be further used to investigate the effects of thermal stabilization on highland barley in order to obtain better products with longer shelf life. Even though this study provides a broader perspective on complicated chemical interactions, further research is required in order to fully comprehend how thermal stabilization affects the qualities of highland barley products. First, the standard sample needs to be used to confirm and measure the volatile compounds. The next are that verification experiments should be carried out. Finally is that the thorough reaction kinetic is needed to be deeply studied to gain more understanding and to regulate a specific reaction. In the future, stabilized technologies should be deeply studied because it is of great significance to the development stable HB product.

## Data availability statement

The original contributions presented in the study are included in the article/supplementary material, further inquiries can be directed to the corresponding author.

## Author contributions

QZ carried out the experiments, wrote the main manuscript, and corrected the manuscript. ZW carried out the experiments and corrected the manuscript. FX corrected the manuscript. GZ conceived the idea and supervised the work. All authors reviewed the manuscript.

## Conflict of interest

The authors declare that the research was conducted in the absence of any commercial or financial relationships that could be construed as a potential conflict of interest.

## Publisher’s note

All claims expressed in this article are solely those of the authors and do not necessarily represent those of their affiliated organizations, or those of the publisher, the editors and the reviewers. Any product that may be evaluated in this article, or claim that may be made by its manufacturer, is not guaranteed or endorsed by the publisher.
